# Inhibitory Effect of Aqueous Extract of Stem Bark of *Cissus populnea* on Ferrous Sulphate- and Sodium Nitroprusside-Induced Oxidative Stress in Rat's Testes *In Vitro*

**DOI:** 10.1155/2013/130989

**Published:** 2013-01-21

**Authors:** Seun F. Akomolafe, Ganiyu Oboh, Afolabi A. Akindahunsi, Ayodele J. Akinyemi, Oluwatosin G. Tade

**Affiliations:** ^1^Department of Biochemistry, Ekiti State University, PMB 5363, Ado-Ekiti, Nigeria; ^2^Department of Biochemistry, Federal University of Technology, PMB 704, Akure, Nigeria; ^3^Department of Biochemistry, Afe Babalola University, PMB 5454, Ado-Ekiti, Nigeria

## Abstract

*Cissus populnea* are plants associated with a myriad of medicinal uses in different parts of the world and are good sources of carotenoids, triterpenoids, and ascorbic acid. The antioxidant properties and inhibitory effect of water extractible phytochemicals from stem bark of *C. populnea* on FeSO_4_ and sodium nitroprusside- (SNP-) induced lipid peroxidation in rat testes were investigated *in vitro*. The results revealed that the extract was able to scavenge DPPH radical, chelate Fe^2+^ and also had a high reducing power. Furthermore, the incubation of the testes tissue homogenate in the presence of FeSO_4_ and SNP, respectively, caused a significant increase in the malondialdehyde (MDA) contents of the testes. However, the aqueous extract of the stem bark of *C. populnea* caused a significant decrease in the MDA contents of both Fe^2+^ (EC_50_ = 0.027 mg/mL) and SNP- (EC_50_ = 0.22 mg/mL) induced lipid peroxidation in the rat testes homogenates in a dose-dependent manner. The water extractible phytochemicals from *C. populnea* protect the testes from oxidative stress and this could be attributed to their high antioxidant activity: DPPH-scavenging ability, Fe^2+^-chelating and -reducing power. Therefore, oxidatively stress in testes could be potentially managed/prevented by this plant.

## 1. Introduction

Infertility is a major clinical problem, affecting people medically and psychosocially. In recent years, oxidative stress has been implicated in the progression of male infertility. Evidence has shown that these damaging events are caused by free radicals [1]. Oxidative stress results from either a decrease of natural cell antioxidant capacity or an increased amount of reactive oxygen species (ROS) in organisms. However, the consumption of foods rich in antioxidant phytochemicals may help fight degenerative diseases caused by oxidative stress by improving body's antioxidant status. 

High levels of Fe play a crucial role in degenerative diseases by acting catalytically in the production of ROS which have the potential to damage cellular lipids, nucleic acids, proteins, and carbohydrate resulting in wide-ranging impairment in cellular function and integrity [2]. ROS can directly attack the polyunsaturated fatty acids of the cell membranes and induce lipid peroxidation. Malondialdehyde (MDA) is the end-product of lipid peroxidation, which is a process where reactive oxygen species (ROS) degrade polyunsaturated fatty acids. 

Recently, much attention has been focused on the role of the antioxidative defense system to combat oxidative stress [3]. Endogenous antioxidants in plants may play an important role in antioxidative defense against oxidative damage [4], possibly preserving the biological functions of cells [5]. There is an increasing interest in the protective biological function of natural antioxidants contained in dietary plants, which are candidates for the prevention of oxidative damage [6]. These antioxidants are polyphenolic compounds, which are found in all plants and in all parts of the plants (tree bark, stalks, leaves, fruits, roots, flowers, pods, and seeds) [6]. 

*Cissus populnea *is a plant associated with a myriad of medicinal uses in different parts of the world. Its extracts have been credited with antibacterial properties [7], as an antitrypanosomal plant and a source of gum powder [8] and as a component of a herbal antisickling Nigerian formula [9]. In Benin Republic, it is used for its diuretic properties while in Ghana it is used as a postharvest ethnobotanical protectant [10]. The aqueous extract of its stem bark is associated with aphrodisiac/fertility potentials among the Yoruba-speaking people of South West Nigeria [11]. The use of *C. populnea*, as an aphrodisiac and fertility enhancer amongst the males, has been attributed to the declining fertility trend that has been established in this population over the years coupled with the attendant increasing levels of erectile dysfunction [12]. Although *C. populnea *has been reportedly used in traditional medicine for the management/prevention of infertility diseases associated with oxidative stress, there is still limited information on some of the possible mechanisms by which they exert this effect. Hence, the objective of this study is to investigate the inhibitory effect of water extractible phytochemicals from stem bark of *C. populnea* on Fe^2+^ and SNP-induced lipid peroxidation in rat's testes *in vitro*.

## 2. Materials and Methods

### 2.1. Sample Collection

Fresh samples of stem bark of *Cissus populnea* were purchased in a local market, in Akure metropolis, Nigeria. Authentication of the plant was carried out in the Department of Biology, Federal University of Technology, Akure, Nigeria. 

### 2.2. Chemicals and Reagents

Chemicals and reagents used such as thiobarbituric acid (TBA), 1,10-phenanthroline, deoxyribose, gallic acid, and Folin-Ciocalteau's reagent were procured from Sigma-Aldrich, Inc., (St. Louis, MO, USA), trichloroacetic acid (TCA) was sourced from Sigma-Aldrich, Chemie GmbH (Steinheim, Germany), dinitrophenyl hydrazine (DNPH) from ACROS Organics (NJ, USA), hydrogen peroxide, methanol, acetic acid, and FeCl_3_ were sourced from BDH Chemicals Ltd., (Poole, England), thiourea, CuSO_4_·5H_2_O, H_2_SO_4_, sodium carbonate, AlCl_3_, potassium acetate, Tris-HCl buffer, sodium dodecyl sulphate, FeSO_4_, and potassium ferricyanide were of analytical grade while the water was glass distilled.

### 2.3. Aqueous Extract Preparation

The sample was washed under running water, air dried after which the dried sample was grinded to powdered form, and kept dry in an air-tight container prior to the extraction. 1 g of the powdered sample was weighed into 20 mL of distilled water and was left for 24 hours [13]. The mixture after 24 hours was filtered and the filtrate centrifuged at 805 ×g for 10 minutes. The clear supernatant collected and was used for subsequent analysis.

### 2.4. Determination of Total Phenolic Content

The total phenolic content was determined using the method reported by Singleton et al. [14]. Appropriate dilutions of the extract were oxidized with 2.5 mL of 10% Folin-Ciocalteau's reagent (v/v) and neutralized by 2.0 mL of 7.5% sodium carbonate. The reaction mixture was incubated for 40 minutes at 45°C, and the absorbance was measured at 765 nm in the spectrophotometer (JENWAY 6305, Barloworld Scientific, Dunmow, UK). The total phenolic content was subsequently calculated and expressed as mg gallic acid equivalent/g dry weight. 

### 2.5. Determination of Total Flavonoid Content

The total flavonoid content of the sample was determined using a slightly modified method reported by Meda et al. [15]. Briefly 0.5 mL of appropriately diluted sample was mixed with 0.5 mL methanol, 50 *μ*L 10% AlCl_3_, 50 *μ*L 1 M Potassium acetate, and 1.4 mL water, and allowed to incubate at room temperature for 30 minutes. The absorbance of the reaction mixture was subsequently measured at 415 nm, and the total flavonoid content was subsequently calculated using quercetin as standard and expressed as mg/g quercetin equivalent dry weight. 

### 2.6. Determination of Vitamin C Content

Vitamin C content of the sample was determined using the method of Benderitter et al. [16]. Briefly, 75 *μ*L DNPH (2 g dinitrophenyl hydrazine, 230 mg thiourea, and 270 mg CuSO_4_·5H_2_O in 100 mL of 5 M H_2_SO_4_) were added to 500 *μ*L reaction mixture (300 *μ*L of an appropriate dilution of the extract with 100 *μ*L 13.3% trichloroacetic acid (TCA) and water). The reaction mixtures were subsequently incubated for 3 h at 37°C, then 0.5 mL of 65% H_2_SO_4_ (v/v) was added to the medium; their absorbance was measured at 520 nm and the vitamin C content of the sample was subsequently calculated using ascorbic acid as standard and expressed as mg/g ascorbic acid equivalent.

### 2.7. Lipid Peroxidation Assay

#### 2.7.1. Experimental Animals

Ten male wistar albino rats weighing between 190 and 250 g were purchased from the Central Animal House, Department of Biochemistry, University of Ilorin, Ilorin, Nigeria. They were housed in stainless steel cages under controlled conditions of a 12 hr light/dark cycle, 50% humidity, and 28°C temperature. The rats were allowed to assess food and water ad libitum. The animals were used in accordance with the procedure approved by the Animal Ethics Committee of the Federal University of Technology, Akure, Nigeria.

#### 2.7.2. Preparation of Tissue Homogenates

The rats were decapitated under mild diethyl ether anaesthesia, and the testes (tissue) were rapidly dissected and placed on ice and weighed. This tissue was subsequently homogenized in cold saline (1/10 w/v) with about 10-up- and downstrokes at approximately 1200 rev/min in a Teflon glass homogenizer (Mexxcare, mc14 362, Aayushi Design Pvt. Ltd. India). The homogenate was centrifuged (KX3400C Kenxin Intl. Co., Hong Kong) for 10 minutes at 3000 ×g to yield a pellet that was discarded and a low-speed supernatant (SI), which was kept for lipid peroxidation assay.

#### 2.7.3. Lipid Peroxidation and Thiobarbibutric Acid Reactions

**Fe^2+^-Induced Lipid Peroxidation**

The lipid peroxidation assay was carried out using the modified method of Ohkawa et al. [17]. Briefly 100 *μ*L of the SI fraction was mixed with a reaction mixture containing 30 *μ*L of 0.1 M pH 7.4 Tris-HCl buffer, extract (100 *μ*L), and 30 *μ*L of 250 *μ*M freshly prepared FeSO_4_ as the prooxidant. The volume was made up to 300 *μ*L by water before incubation at 37°C for 2 hrs. The color reaction was developed by adding 300 *μ*L 8.1% SDS (sodium dodecyl sulphate) to the reaction mixture, this was subsequently followed by the addition of 500 *μ*L of acetic acid/HCl (pH 3.4) mixture and 500 *μ*L of 0.8% thiobarbituric acid (TBA). This mixture was incubated at 100°C for 1 hr. Thiobarbituric acid reactive species (TBARS) produced were measured at 532 nm and expressed as malondialdehyde (MDA) produced (% control) using MDA standard curve (0–0.035 mM).

Also, for sodium nitroprusside- (SNP-) induced lipid peroxidation, the procedure was carried out as above using 5 mM sodium nitroprusside (SNP) as the prooxidant. 

#### 2.7.4. DPPH-Free Radical Scavenging Ability

The free radical scavenging ability of the extracts against DPPH (1,1-diphenyl-2 picrylhydrazyl) free radical was evaluated as described by Gyamfi et al. [18]. Briefly, an appropriate dilution of the extract (1 mL) was mixed with 1 mL of 0.4 mM methanolic solution containing DPPH radicals, the mixture was left in the dark for 30 min, and the absorbance was measured at 516 nm. The control was carried out using 2 mL DPPH solution without the test samples. The percentage (%) of DPPH-free radical scavenging ability was subsequently calculated as follows:

(1) DPPH  scavenging  ability(%) =[(AbsControl−AbsSamples)AbsControl]×100.

#### 2.7.5. Fe^2+^ Chelation Assay

The Fe^2+^ chelating ability of the extract was determined using a modified method of Minotti and Aust [19] with a slight modification by Puntel et al. [20]. Freshly prepared 500 *μ*M FeSO_4_ (150 *μ*L) was added to a reaction mixture containing 168 *μ*L of 0.1 M Tris-HCl (pH 7.4), 218 *μ*L saline and the extracts (0–25 *μ*L). The reaction mixture was incubated for 5 min, before the addition of 13 *μ*L of 0.25% 1,10-phenanthroline (w/v). The absorbance was subsequently measured at 510 nm in a spectrophotometer. The Fe (II) chelating ability was subsequently calculated with respect to the reference (which contains all the reagents without the test sample).

#### 2.7.6. Determination of Reducing Property

The reducing property of the extract was determined by assessing the ability of the extract to reduce FeCl_3_ solution as described by Oyaizu [21]. A 2.5 mL aliquot was mixed with 2.5 mL of 200 mM sodium phosphate buffer (pH 6.6) and 2.5 mL of 1% potassium ferricyanide. The mixture was incubated at 50°C for 20 min, and then 2.5 mL of 10% trichloroacetic acid was added. This mixture was centrifuged at 650 rpm for 10 min. 5 mL of the supernatant was mixed with an equal volume of water and 1 mL of 0.1% ferric chloride. The absorbance was measured at 700 nm. The ferric reducing antioxidant property was subsequently calculated using ascorbic acid as standard.

#### 2.7.7. Data Analysis

The results of the replicates were pooled and expressed as mean ± standard deviation. Analysis of variance and Student's *t*-test were carried out [22]. Significance was accepted at *P* ≤ 0.05.

## 3. Results and Discussion

Many plants are rich sources of phytochemicals, and intakes of these plant chemicals have protective potential against degenerative diseases [23]. The total phenolic content and total flavonoid content of stem bark of *C. populnea* are presented in Table 1. The total phenolic content of the plant (17.33 mg/g) is lower than what was reported for some hot peppers and green teas [24], but higher than some tropical leafy vegetables [25, 26]. Phenolic compounds can protect the human body from free radicals, whose formation is associated with the normal metabolism of aerobic cells. They are strong antioxidants capable of removing free radicals, chelate metal catalysts, activate antioxidant enzymes, reduce alpha-tocopherol radicals, and inhibit oxidases [27]. 

The total flavonoid content of *C. populnea* (0.059 mg/g) is lower than what was reported for some tropical green leafy vegetables [28]. The presence of derivatives of flavonoids has been found in many herbs; moreover, numerous studies have conclusively shown that the majority of the antioxidant activity may be from compounds such as flavonoids, isoflavones, flavones, anthocyanins, catechin, and isocatechin rather than from vitamins C, E and *β*-carotene [29, 30]. Flavonoids have antioxidant activity and could therefore lower cellular oxidative stress [30]. Polyphenols are considered to be strong antioxidants due to the redox properties of their hydroxyl groups [31]. 

The vitamin C content of stem bark of *C. populnea* is presented in Table 1. Vitamin C has been reported to contribute to the antioxidant activities of plant food. Ascorbic acid is a good reducing agent and exhibits its antioxidant activities by electron donation [25, 26]. It helps the immune system to fight off foreign invaders and tumor cells and supports the cardiovascular system by facilitating fat metabolism and protecting tissues from free radical damage, and it assists the nervous system by converting certain amino acids into neurotransmitters. As a water-soluble antioxidant, vitamin C is in a unique position to “scavenge” aqueous peroxyl radicals before these destructive substances damage the lipids [32]. The vitamin C content of the plant is higher than that of related spices such as basil, bird pepper, black pepper, cinnamon, nutmeg, oregano, parsley and rosemary [33], and some commonly consumed green leafy vegetables in Nigeria [25, 26], green and red pepper, and some commonly consumed and underutilized tropical legumes [34]. It is also worth noting that the vitamin C content of the plant is higher than its total phenol content. The high vitamin C content of the plant will definitely contribute additively or synergistically to the observed antioxidant and medicinal properties of the plant.

**Figure 1:**
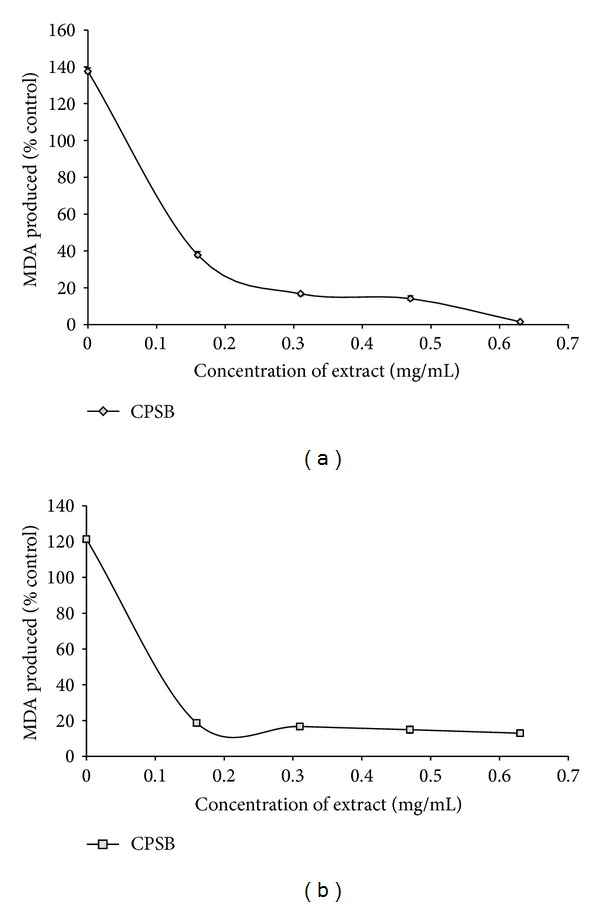
(a) Inhibition of Fe^2+^-induced lipid peroxidation in rat testis by aqueous extract of stem bark of *Cissus populnea. *(b) Inhibition of SNP-induced lipid peroxidation in rat testis by aqueous extract of stem bark of *Cissus populnea. *

Lipids are considered to be the most susceptible macromolecules and are present in male reproductive organ. One of the byproducts of lipid peroxidation is malondialdehyde, this byproduct has been used in various biochemical assays to monitor the degree of peroxidative damage sustained by spermatozoa [35, 36]. Testicular toxicity due to Fe^2+^ impairs fertility [37]. The finding that Fe^2+^ caused a significant increase in the MDA content of the testis homogenate agreed with earlier report where Fe^2+^ was shown to be a potent initiator of lipid peroxidation [14]. The increased lipid peroxidation in the presence of Fe^2+^ could be attributed to the fact that Fe^2+^ can catalyze one-electron transfer reactions that generate reactive oxygen species, such as the reactive OH^∙^, which is formed from H_2_O_2_ through the Fenton reaction. Iron also decomposes lipid peroxides, thus generating peroxyl and alkoxyl radicals, which favors the propagation of lipid oxidation [38]. In the testis, Fe-induced lipid peroxidation destroys the structure of lipid matrix in the membranes of spermatozoa, and it is associated with loss of motility and impairment of spermatogenesis [37]. Therefore, possible depletion of iron could decrease oxidative stress throughout the whole body [39]. The ability of the water extractible phytochemicals from stem bark of *C. populnea* to inhibit Fe^2+^-induced lipid peroxidation in the testes homogenate is presented in Figure 1. The result revealed that the incubation of the testes homogenate in the presence of Fe^2+^ caused a significant (*P* < 0.05) increase in the MDA content (137.5%) of the rat testes homogenate when compared with the basal (100%). However, the aqueous extract of stem bark of *C. populnea* inhibited MDA production in rat's testes in a dose-dependent manner (0–0.63 mg/ml). Nevertheless, judging by the EC_50_ (extract concentration causing 50% inhibition) values in Table 2, the plant (EC_50_ = 0.027 mg/ml) had a significantly (*P* < 0.05) high inhibitory effect on Fe^2+^-induced lipid peroxidation in the testes homogenate. The decrease in the Fe^2+^-induced lipid peroxidation in the rat testes homogenates in the presence of the extract could be as result of the ability of the extracts to chelate Fe^2+^ and/or scavenge free radicals produced by the Fe^2+^-catalyzed production of reactive oxygen species (ROS) in the rat testes. 

**Figure 2:**
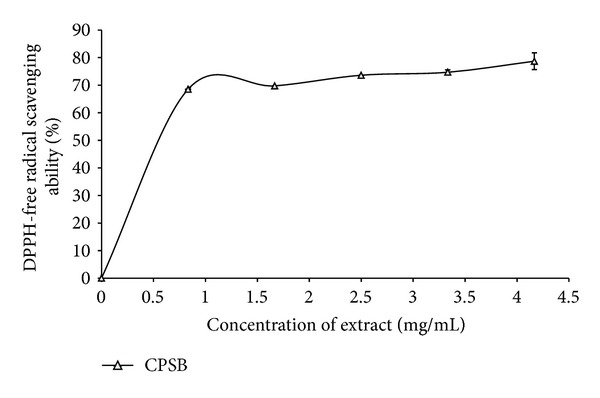
DPPH radical scavenging ability of aqueous extract of stem bark of *Cissus populnea. *

Antioxidants carry out their protective role on cells either by preventing the production of free radicals or by neutralizing/scavenging free radicals produced in the body or by reducing/chelating the transition metal composition of foods [27, 40]. In an attempt to explain the main mechanism through which the water extractable phytochemicals in the plant extract protect testes tissue against Fe^2+^-induced lipid peroxidation, the DPPH radical scavenging and Fe^2+^-chelating abilities were assessed. The prevention of the chain initiation step by scavenging various reactive species such as free radicals is considered to be an important antioxidant mode of action [41]. DPPH is a free radical donor that accepts an electron or hydrogen to become a stable diamagnetic molecule [42]. The tendencies of electron or hydrogen donation are critical factors in characterizing antioxidant activity that involves free radical scavenging [43]. Foods of plant origin usually contain natural antioxidants that can scavenge free radicals [6]. These antioxidants are polyphenolic compounds which have protective effect against diseases [6] and can be found in all plants and in all parts of the plants (tree bark, stalks, leaves, fruits, roots, flowers, pods, and seeds) [6]. The DPPH radical scavenging ability of the aqueous extract from *C. populnea* as represented in Figure 2 revealed that the extract scavenged DPPH radicals in a dose-dependent pattern (0–3.33 mg/mL). 

The plant extracts also chelate Fe^2+^ (Table 3). This result, however, is in agreement with the Fe^2+^-induced lipid peroxidation (Figure 1), phenolic content (Table 1), and antioxidant activity of the extracts, suggesting that Fe chelation may be one of the possible mechanisms through which antioxidant phytochemicals from stem bark of *C. populnea* prevent lipid peroxidation in tissue by forming a complex with Fe, thus preventing the initiation of lipid peroxidation. 

Furthermore, the reducing power of the extractable phytochemicals from *Cissus populnea* (stem bark) expressed as ascorbic acid equivalent (AAE) is presented in Table 3. The reducing power as typified by the ability of the plant extracts to reduce Fe^3+^ to Fe^2+^ is a potent antioxidation defense mechanism, and two mechanisms available to affect this reducing power is by electron transfer and hydrogen atom transfer [28]. Allhorn et al. [44] reported that the reducing property can be a novel antioxidant defense mechanism, possibly through the ability of the antioxidant compound to reduce transition metals. Therefore, the higher reducing ability of *Cissus populnea* extract may have contributed to the higher protective effect observed. 

Likewise, the incubation of rat's testes tissue homogenates in presence of sodium nitroprusside also caused a significant increase (*P* < 0.05) in the rat's testes malondialdehyde (MDA) content as shown in Figure 1(b); however, the extract inhibited MDA production content in the tissue in a dose-dependent (0–0.63 mg/ml). However, judging by the EC_50_ value in Table 2, the aqueous extract of *C. populnea* had a high inhibitory effect on sodium nitroprusside-induced lipid peroxidation in rat testes. The protective properties of the plant extract against sodium nitroprusside-induced lipid peroxidation in the testes could be because of the ability of the antioxidant phytochemicals present in the aqueous extract to quench-scavenge the nitrous radical and Fe produced from the decomposition of sodium nitroprusside.

## 4. Conclusion

The aqueous extract of stem bark of *C. populnea* was able to protect the testes homogenate from both Fe^2+^- and SNP-induced lipid peroxidation* in vitro*. A part of the mechanisms through which the water extractable phytochemicals in the plant protect the testes from oxidative stress may be through their phenolic content and antioxidant activity; DPPH-scavenging ability, Fe^2+^-chelating and -reducing power. Therefore, oxidatively stress testes could be potentially managed/prevented by this plant. 

## Figures and Tables

**Table 1:** Total phenolic, flavonoid, and vitamin C content of stem bark of *Cissus populnea *(stem bark).

Phenolic content (mg GAE/g)	Flavonoid content (mg QUE/g)	Vitamin C (mg AAE/g)
17.33 ± 0.00	0.059 ± 0.01	22.06 ± 0.37

Values represent means ± standard deviation of triplicate readings.

GAE: gallic acid equivalent, QUE: quercetin equivalent, and AAE: ascorbic acid equivalent.

**Table 2:** EC_50_ (extract concentration causing 50% inhibition) values of Inhibition of Fe^2+^- and SNP-induced lipid peroxidation in rat testis by aqueous extract of *Cissus populnea *(stem bark).

EC_50_ (mg/mL) Fe^2+^- induced lipid peroxidation	EC_50_ (mg/mL) SNP- induced lipid peroxidation
0.027 ± 0.01	0.22 ± 0.00

Values represent mean ± standard deviation, number of samples *n* = 3.

**Table 3:** Fe^2+^-chelating ability and Ferric reducing antioxidant properties (FRAP) of aqueous extract of *Cissus populnea *(stem bark).

Fe^2+^ chelating ability (%)	FRAP (%)
93.25 ± 0.81	11.52 ± 0.04

Values represent mean ± standard deviation, number of samples *n* = 3.
